# Perceived stress and musculoskeletal pain are prevalent and significantly associated in adolescents: an epidemiological cross-sectional study

**DOI:** 10.1186/s12889-015-2414-x

**Published:** 2015-10-23

**Authors:** Berit Østerås, Hermundur Sigmundsson, Monika Haga

**Affiliations:** Department of Physiotherapy, Faculty of Health Education and Social Work, Sør-Trøndelag University College, Tungasletta 2, 7004 Trondheim, NORWAY; Department of Psychology, Faculty of Social Sciences and Technology Management, Norwegian University of Science and Technology (NTNU), Trondheim, NORWAY

**Keywords:** Adolescents, Perceived stress, Musculoskeletal pain, Stress-pain associations, Stress-related pain

## Abstract

**Background:**

Long-term musculoskeletal pain and negative stress are health risks with adverse long-term health effects, and these health risks seem to increase among young people. The mechanisms behind this are unclear. There is a need for a better understanding of perceived stress and musculoskeletal pain among adolescents, in order to improve health promotion and treatment approaches in this group.

**Methods:**

Objectives were to evaluate the current prevalence of perceived stress and musculoskeletal pain in 15 and 16 year olds, to explore stress-pain associations and the probability that perceived stress (PSQ) was related to the reporting of pain and variations in pain, and to investigate possible differences in stress between different types of musculoskeletal pain in the adolescents. A cross-sectional study was conducted. Elementary schools participated. The outcomes were stress (Perceived stress questionnaire; PSQ) and musculoskeletal pain (pain/no pain, pain sites, pain duration and pain intensity (Visual analogue scale; VAS).

**Results:**

Fifty-one point two percent (*N* = 422) reported pain, of which 70.8 % reported long-term pain. Some more girls (57.9 %) reported pain. 22.0 % of the study population reported moderate to severe stress (PSQ ≥ 0.45), of which 79.6 % were bothered by pain (Pearson Chi-square 38.47, *p* ≤ .001). All stress and pain variables were significantly associated (*p* < .01). The strongest association appeared between pain intensity (VAS) and stress (PSQ) (*r* = 0.40). Perceived stress (PSQ) was associated with the reporting of pain among the adolescents (Odds Ratio [OR] 1.68) and could explain some of the variation in pain intensity (VAS; β = 0.15, *p* < .001) and number of pain sites (β = 0.14, *p* < .01), according to the regression analyses. There were no mean differences in stress (PSQ) between different types of musculoskeletal pain.

**Conclusions:**

There was high prevalence of musculoskeletal pain, long-term pain and moderate to severe stress (PSQ ≥ 0.45) in this study sample. Perceived stress (PSQ) was related to the reporting of musculoskeletal pain among the adolescents and could explain some of the variation in pain intensity (VAS) and number of pain sites. There were no differences in stress levels (PSQ) between different types of musculoskeletal pain in the adolescents.

**Electronic supplementary material:**

The online version of this article (doi:10.1186/s12889-015-2414-x) contains supplementary material, which is available to authorized users.

## Background

Several authors have characterized adolescence as a stressful, transiting stage of life [1–3], wherein pain experiences are common. [4–6] As pointed out by Ursin and Erikson [7] and Del Giudice et al. [1], stress is primarily positive and a normal activation response, leading to adaptation and change. However, the positive and functional role of stress might be diminished or entirely lost, when the stress response is detached from the stress stimuli, prolonged and sensitized [1, 8, 9]. This corresponds to the nature of pain. Pain is primarily positive (meaningful) and necessary for survival, but can lose its appropriate and positive function when it becomes long-term and sensitized [10]. Then, pain turns into a disabling and exhausting phenomena and might cause excessive stress and more pain [10, 11].

Population-based studies have found high prevalence of musculoskeletal pain in adolescents [12–15]. The young HUNT study 2008 reported chronic pain in 44.4 % of the adolescents (aged 13–18) [6] and multisite pain in 8.8–31.0 % [16]. In the young Swedish population there is an increase in neck and shoulder pain, which is described as a psychosocial stress-related phenomena [12, 13] Several authors have related neck, shoulder and back pain to stress and psychosocial factors, also in adolescents and children [3–5, 17–19]. In general, there appears to be an association between musculoskeletal pain and psychological factors in young people [15, 20].

There are several levels of stress-pain interactions, which can be termed neurophysiological, psychophysiological, cognitive-behavioral, and genetic-behavioral levels. The neurophysiological level explains how pain induces a stress response in the “supersystem”, i.e., the interconnected nervous system, endocrine system and immune system [21–23]. The psychophysiological level includes the impact of emotions and negative stress on the supersystem and pain perception [21, 22]. The cognitive-behavioral level explains how appraisals of danger and coping capability (i.e., perceived stress) and choice of behavior (exposure, fear-avoidance; adaptive/ mal-adaptive) are transmitted by supersystem-activation and hence influence the pain experience [8, 21–23]. The genetic-behavioral level describes individual differences in the susceptibility of stress and pain due to differences in genotypes and epigenetic factors [1, 21, 24, 25].

A modified transactional model of stress explains how several factors contribute in the stress perception process on a cognitive-behavioral level, including personal aspects, stress exposures and reactions [26, 27]. Health consequences to stress depends on the individual appraisal of available resources under the influence of personality characteristics, according to this stress model [26, 27]. Earlier stressful life experiences seem to predict the current stress response and coping in adolescents, and can make young people vulnerable to actual life challenges and threaten their long-term health [28–30].

Negative stress and prolonged pain are health risks with adverse long-term health effects [11, 22]. Previous studies have demonstrated high prevalence of musculoskeletal pain and different psychosocial factors in adolescents [6, 13, 15, 20]. There is however still lacking knowledge about the stress-pain relationship on a behavioral level in adolescents, which might illuminate possible mechanisms behind the development of illness among young people [31–35]. The main objectives of this work were to examine perceived stress and musculoskeletal pain in 16 year old adolescents with respect to 1) prevalence, 2) different stress-pain associations, 3) the probability that perceived stress (PSQ) was associated with the reporting of pain and variation in pain, and 4) possible differences in mean stress (PSQ) between different types of musculoskeletal pain.

## Method

### Participants

This study’s cross-sectional sample included pupils in 10th grade (in their 16th or 17th year) in public elementary schools in the Trondheim municipality in Norway. Of a total of 17 schools, six agreed to participate. The schools varied in size and localization (from city to suburb), admitting pupils with different sociocultural- and economic backgrounds [36], and was representative of Norwegian 10th grade pupils. The data were collected during spring and autumn 2013.

### Procedure

The data collection process was approved by the Regional Committee for Medical Research Ethics in Trondheim, and the study was in line with the Declaration of Helsinki [37]. The manuscript reporting adheres to STROBE guidelines for reporting observational Research (Additional file 1). The purpose of the study was explained in detail to the school principals and described in the invitation to participants and in the introduction of each questionnaire (Additional file 2), emphasizing the voluntary and anonymous participation. The respective principals from each of the schools approved the content of the questionnaire prior to agreeing to participate in the survey. Passive consent from the participants was found to be sufficient according to the Regional Committee for Medical Research Ethics, because no sensitive personally identifying data were collected. The pupils accepted the invitation to participate by answering the questionnaire. The pupils’ answers were enclosed inside the questionnaire folder, thus invisible to the administrators (principals or teachers) who collected and returned the questionnaires in concealed envelopes. Questionnaire administration was completed in one session, in whole class groups, during one regular school period of 45 min.

### Study outcomes

Perceived stress and musculoskeletal pain were outcome variables*.*

#### Stress

Stress was measured by the Perceived stress questionnaire (PSQ) [26, 38]. PSQ is a valid instrument for measuring perceived stress [27, 39] in an adolescent population [40]. The PSQ includes 30 items that are assigned to four factors: worries, tension, (lack of) joy, and demands [29, 41]. The responses to the items in the PSQ refers to experiences in the previous month, and each item is scored on a 4-point rating scale (1 = almost never, 2 = sometimes, 3 = often and 4 = usually). The resulting PSQ Total Score is linearly transformed between 0 and 1 according to Levenstein et al. [26]; PSQ = (raw value-30)/90. The cut-off score for moderate to severe stress was set to ≥0.45 (higher stress). This was based on the mean stress score (total PSQ) in the present study population (0.33, standard deviation [SD] 0.16), which corresponds to previous studies using PSQ in population surveys [27, 41]. For the purpose of regression analyses, the PSQ-index was transformed to a 0–10 scale, termed the modified PSQ (mod.PSQ). Previous studies have shown a high Cronbach’s α value of the PSQ (α = 0.93) [27]. The authors of the PSQ granted us permission for translation and back translation of the PSQ, and authorized our final version.

#### Pain

Pain was measured by a dichotomous variable (bothered by pain/ not bothered by pain), questions about localization (pain site), duration, and intensity. Pain site was divided into six main categories, similar to the questionnaire in the Young-HUNT Study 2008 [16, 20]; head, neck, shoulder, back, arm, lower extremity, with an open line for additional sites. The six main pain sites were allocated into four groups for analytic purposes. Group one comprised head and eventually other pain sites. Group two comprised neck, shoulder and eventually other pain sites, excluding the head. Group three comprised back and eventually extremities (arm and/or lower extremity), but excluded head and neck and shoulder. Group four comprised extremity pain exclusively (arm and/ or lower extremity). Duration was divided into five categories; 0–2 weeks, 2–4 weeks, 1–2 months, 2–3 months, 3 months or more, scored 1–5. The latter pain duration category, i.e., 3 months or more was termed long-term pain, in accordance with the IASP Classification of Chronic Pain [42] regarding musculoskeletal pain. Intensity was measured by a Visual analogue scale (VAS: 0–10 cm) [43] as the average pain during the last week, with 0 indicating “no pain” and 10 “worst pain imaginable”. The VAS is reliable as a measure of pain intensity in children [44] and is used to detect differences in pain between groups of adolescents [45]. The pain questionnaire in this study also included two questions about injuries and/ or diseases which could possibly be related to the pain experience.

Demographic data included gender, height and weight. Height and weight were transformed into a body mass index (BMI) variable, which was included in the analyses. BMI is considered relevant for stress and pain, also in adolescents [46, 47].

### Statistical analysis

All data are analyzed with IMB SPSS statistics 21. Cronbach’s α was computed to estimate the internal consistency of the PSQ. Descriptive analyses including means and standard deviations for the continuous variables were calculated, separately for pain/ no pain. Person Chi-square was used in the 2×2-table of the dichotomous variables pain/ no pain and gender. Pearson product-moment correlation (r) was used to test bivariate associations between the continuous variables. A bivariate logistic regression was performed to evaluate the probability that perceived stress (mod. PSQ) was associated with the reporting of pain among the adolescents, applying gender and BMI as covariates. Linear regression analyses evaluated the probability that pain duration, perceived stress (mod.PSQ), gender and BMI could explain variation in pain intensity (VAS) and number of pain sites, with the latter two variables applied as dependent variables. General Linear Models (GLM’s) were used for between pain site group analyses, with adjustments for gender and BMI. Total stress (PSQ) and different factors of stress (worries, tension, joy, and demands) as well as pain duration and pain intensity (VAS) were applied as dependent variables, while gender and BMI were included as covariates. The adjustments for multiple comparisons were performed by using Bonferroni. To evaluate the strength of pain site group/category on the dependent variables, effect sizes were calculated. Effect size (Cohen’s d) is defined as the difference in outcome between the groups divided by the standard deviation of the baseline scores for this outcome [48]. Cohen [49] has presented some guidelines for the strength of effects: small (.20), medium (.50) and large (.80+). *p*-values ≤ .05 was considered statistically significant.

## Results

430 questionnaires were distributed. The number of completed questionnaires returned was *N* = 423, giving an overall response rate of 98.4 %; 218 (51.5 %) girls and 204 (48.2 %) boys, and one participant did not report gender.

There was a small number of missing data. One participant did not report gender and one did not report on pain/ no pain. Two participants reported to have pain without specifying pain site. Mainly data from the items on page two in the questionnaire (the only back page) were exposed for missing. Apparently some of the participants overlooked these items, resulting in 18 missing responses on pain intensity (VAS), and 19 missing responses on pain duration. The participants with missing data were excluded listwise in the analyses.

Descriptive analyses are outlined in Tables 1 and 2. Study variables are displayed for the study sample (*N* = 422) in general, as well as separately for pain (*N* = 216)/ no pain (*N* = 206). More than half of adolescents (51.2 %) reported to be bothered by musculoskeletal pain (*n* = 216), of which 70.8 % (*n* = 153) reported long-term pain. More girls (57.9 %) than boys reported to be bothered by pain (Pearson Chi-square 7.11, *p* ≤ .05). The prevalence of moderate to severe stress (PSQ ≥ 0.45) was 22.0 % (*n* = 93). Most of the participants reporting moderate to severe stress (PSQ ≥ 0.45) also were bothered by musculoskeletal pain (*n* = 73, Pearson Chi-square 38.47, *p* ≤ .001). The modified PSQ-index mean score in the study sample was 3.3. (SD 1.6). The group of participants with pain (*n* = 216; “pain group”) presented with higher mean scores for all stress variables than the group without pain, with highest mean scores for demands (4.9 [SD 2.2]) and tension (4.2 [SD 1.9]).

**Table 1:** Study variables; prevalence separately for pain/ no pain

		Pain	No pain	Pearson
	*N* = 422	*N* = 216	*N* = 206	Chi-square
Girls	218 (51.7 %)	125 (57.9 %)	92 (44.6 %)	
Boys	204 (48.3 %)	91 (42.1 %)	113 (54.9 %)	7.11*
Pain	216 (51.2 %)			
Long-term pain (3 months and more)	153 (36.3 %)			
Stress				
Higher stress (PSQ ≥ 0.45)	93 (22.0 %)	74 (34.3 %)	19 (9.2 %)	38.47**
Lower stress (PSQ < 0.45)	329 (78 %)	142 (65.7 %)	187 (90.8 %)	
Total (mod.PSQ; 0–10), mean (SD)	3.3 (1.6)	3.9 (1.7)	2.7 (1.3)	
Worries (0–10), mean (SD)	2.7 (1.9)	3.3 (2.1)	2.1 (1.5)	
Tension (0–10), mean (SD)	3.5 (1.8)	4.2 (1.9)	2.8 (1.3)	
Joy (0–10), mean (SD)	3.6 (1.9)	4.0 (2.0)	3.1 (1.7)	
Demands (0–10), mean (SD)	4.2 (2.1)	4.9 (2.2)	3.5 (1.8)	
BMI	20.8 (3.1)	21.0 (3.1)	20.5 (3.2)	

*PSQ* Perceived stress questionnaire

*p ≤ .05; ** ≤ 001

**Table 2:** Pain study variables

	*N* = 216
Pain	
Long-term pain (3 months and more)	153 (70.8 %)
Head pain	76 (35.2 %)
Neck pain	40 (18.5 %)
Shoulder pain	40 (18.5 %)
Back pain	69 (31.9 %)
Arm pain	22 (10.2 %)
Lower extremity pain	112 (51.9 %)
Sites (0–6), mean (SD)	1.7 (1.1)
Duration (1–5), mean (SD)	3.3 (1.3)
Intensity (VAS; 0–10), mean (SD)	4.5 (2.4)

The most prevalent pain sites were lower extremity (*n* = 112), head (*n* = 76) and back pain (*n* = 69) (Table 2). Mean (SD) pain intensity (VAS; 0–10) in the “pain group” was 4.5 (SD 2.4). Descriptive analyses of mean (SD) stress and pain variables in the four different pain site groups are presented in Table 3. The table shows highest stress and pain values in the head pain group except for worries, which was slightly higher in the back pain group.

**Table 3:** Mean (SD) of primary outcome variables in the different pain site groups

Pain site groups							
	Stress					Pain	
	(mod.PSQ)	Worries	Tension	Joy	Demands	Duration	Intensity (VAS)
Head (*n* = 76)	4.24 (1.84)	3.55 (2.22)	4.53 (1.88)	4.43 (1.65)	5.36 (2.42)	3.37 (1.37)	4.77 (2.43)
Neck and shoulder (*n* = 36)	3.73 (1.73)	3.11 (1.99)	4.29 (2.04)	3.91 (2.23)	4.52 (1.84)	3.25 (1.14)	4.27 (2.25)
Back (*n* = 34)	3.96 (1.54)	3.58 (2.05)	4.06 (2.00)	3.94 (2.02)	4.74 (2.0)	3.35 (1.25)	4.38 (2.56)
Extremities (*n* = 61)	3.29 (1.64)	2.82 (1.85)	3.79 (1.79)	3.50 (1.90)	4.33 (1.96)	3.21 (1.34)	3.92 (2.27)

*mod.PSQ* Modified Perceived stress questionnaire- index

The PSQ showed a Cronbach’s α of 0.90. Correlation analyses of the continuous variables are presented in Table 4, showing significant (*p* < .01) correlations between all variables concerning stress and pain, as well as between BMI and pain intensity (VAS), and joy. The highest stress-pain correlations involved pain intensity (VAS); i.e., between VAS and total stress (PSQ) (*r* = 0.40) and between VAS and tension (*r* = 0.39). Pain intensity (VAS) also showed strong correlations to number of pain sites (*r* = 0.63) and pain duration (*r* = 0.69). BMI correlated weakly with joy and pain intensity (VAS) (Table 4).

**Table 4:** Correlations between the pain and stress study variables

	Pain sites	Pain duration	Pain intensity	Total	W	T	J	D	BMI
			VAS	PSQ					
Pain									
Sites	1								
Duration	0.59**	1							
Intensity (VAS)	0.63**	0.69**	1						
Stress									
Total PSQ	0.35**	0.34**	0.40**	1					
Worries (W)	0.29**	0.28**	0.34**	0.94**	1				
Tension (T)	0.37**	0.36**	0.39**	0.84**	0.70**	1			
Joy (J)	0.22**	0.25**	0.30**	0.67**	0.53**	0.50**	1		
Demands (D)	0.30**	0.25**	0.31**	0.79**	0.69**	0.61**	.37**	1	
BMI	0.19	0.49	0.12*	0.02	0.04	0.01	0.12*	−0.05	1
Cronbach’s α				0.90					

*VAS*Visual analogue scale, *PSQ* Perceived stress questionnaire, *W* Worries, *T* Tension, *J* Joy, *D* Demands

**p < .01

All the regression analyses are summarized in Tables 5 and 6. The logistic regression analyses revealed that perceived stress (mod. PSQ) was associated (*p* < .001) with the reporting of musculoskeletal pain among the adolescents, yielding an Odds Ratio (OR) of 1.68 (Tables 5 and 6). The full regression model explained between 14.4 % (Cox & Snell R Square) and 19.2 % (Nagelkerke R Square) of the variance in the pain variable, correctly classifying 66.1 % of the cases. The test for model fit yielded support for the full model with *χ*2 = 56.34 (*p* < .001) and a non-significant Hosmer and Lemeshow test (*p* = 0.875).

**Table 5:** Summary of the regression analyses

Results of logistic regression analyses evaluating the probability that perceived stress was associated with the reporting of pain (the dependent dichotomous variable pain/no pain)				
	B	95 % CI for Odds Ratio		
		Lower	Odds	Upper
Constant	−2.51			
Stress (mod.PSQ; 0–10)	0.52***	1.42	1.68	1.99

Control variables: gender and BMI ***p < .001

**Table 6:** Summary of the regression analyses

Results of the linear regression analysis evaluating the probability that perceived stress could explain variation in pain intensity (VAS)					
	В	SE B	β	F	R^2^
Constant	−2.78	0.74			
Pain duration (1–5)	1.21	0.08	0.64***		
Stress (mod.PSQ; 0–10)	0.25	0.07	0.15***		
Gender	−0.19	0.22	−0.03		
BMI	0.07	0.03	0.09*	93.31***	0.52
Results of the linear regression analysis evaluating the probability that perceived stress could explain variation in number of pain sites					
Constant	0.68	0.35			
Pain duration (1–5)	0.43	0.04	0.55***		
Stress (mod.PSQ; 0–10)	0.10	0.03	0.14**		
Gender	0.01	0.11	0.0		
BMI	0.01	0.02	0.03	51.67***	0.37

*CI* Confidence interval, *VAS* Visual analogue scale, *mod.PSQ* Modified Perceived stress questionnaire-index

*p < .05; **p < .01; ***p < .001

The linear regression analysis with pain intensity (VAS) as dependent variable, revealed that pain duration (β = 0.64, *p* < .001) and perceived stress (PSQ, β = 0.15, *p* < .001) explained some of the variation in pain intensity (VAS) (Tables 5 and 6). BMI demonstrated a weak explanatory effect (β = 0.09 *p* < .05). Gender did not explain any variation in pain intensity (VAS), according to the regression analyses. The regression model explained 52.3 % of the variation in the pain intensity variable (VAS). The R^2^ (0.523) and significant F-value (93.31, *p* < .001) supported good fit of the model. The adjusted R^2^ (0.518) indicated very sparse loss of power. A Durbin-Watson test value close to two (1.70) indicated independence of the residuals. The linear regression analysis with pain sites as dependent variable, revealed that pain duration (β = 0.55, *p* < .001) and perceived stress (PSQ; β = 0.14, *p* < .01) also explained some of the variation in the number of pain sites (Tables 5 and 6). Neither gender nor BMI seemed to explain variation in this pain variable. This regression model explained 37.7 % of the variation in number of pain sites. The R^2^ (0.377) and significant F-value (51.67, *p* < .001) supported good fit of the model. The adjusted R2 (0.370) indicated limited loss of power. A Durbin-Watson test value close to two (1.97) indicated independence of the residuals.

Between pain site groups comparison analyses (Table 7) revealed no significant adjusted mean differences with respect to the included stress and pain variables (which are presented with the greatest differences in means in Table 3). Effect size calculations for the pairwise comparisons showed small to medium effects between head pain and extremity pain groups, and between back pain and extremity pain groups. The greatest effect size [0.5] appeared between head pain and extremity pain groups with respect to joy (Table 7).

**Table 7:** Adjusted mean (95 % CI) difference between pain site groups with corresponding effect size

Pain site groups		Adjusted mean difference between groups^a^ (95 % CI) [effect size]			
		Tension	Joy	Demands	Pain intensity (VAS)
Head	Neck and shoulder	0.20 (−0.80–1.19) [0.1]	0.64 (−0.48–1.74) [0.3]	0.68 (−0.52–1.88) [0.3]	0.40 (−0.79–1.60) [0.2]
	Back	−0.05 (−1.1–1.0) [−0.0]	0.60 (−0.58–1.78) [0.3]	0.13 (−1.15–1.41) [0.1]	0.09 (−1.18–1.35) [0.0]
	Extremities	0.60 (−0.29–1.48) [0.3]	0.87 (−0.12–1.86) [0.5]	0.86 (−0.22–1.93) [0.4]	0.71 (−0.35–1.77) [0.3]
Neck, shoulder	Back	−0.25 (−1.42–0.91) [−0.1]	−0.03 (−1.34–1.27) [−0.0]	−0.55 (−1.97–0.86) [−0.3]	−0.32 (−1.73–1.09) [−0.1]
	Extremities	0.40 (−0.63–1.43) [0.2]	0.23 (−0.92–1.39) [0.1]	0.17 (−1.07–1.43) [0.1]	0.31 (−0.94–1.55) [0.1]
Back	Extremities	0.65 (−0.42–1.73) [0.4]	0.27 (−0.94–1.47) [0.1]	0.73 (−0.58–2.04) [0.4]	0.63 (−0.67–1.92) [0.3]

No statistical significant mean differences between groups

*CI* Confidence interval

^a^The mean differences between groups were adjusted for gender and BMI

The responses concerning injuries and/ or diseases possibly related to the pain experience were very divergent and difficult to categorize for analytic purposes. To avoid group level bias due to individual interpretations, these items were excluded in the subsequent analysis.

## Discussion

The objectives in this study were to examine perceived stress in relation to musculoskeletal pain in adolescents; the prevalence, associations, stress as a possible explanatory factor of pain and variation in pain, and possible differences in mean stress (PSQ, worries, tension, joy, demands) between different types of musculoskeletal pain.

The findings demonstrated a high prevalence of musculoskeletal pain (51.2 %) and long-term musculoskeletal pain (36.3 %) in the adolescents. This supports findings from the Young-HUNT Study 2008 [6] and confirms that musculoskeletal pain is common and normal to experience during adolescence [50]. Lower extremity pain was the pain site most frequently reported, followed by head and back pain (Table 2). The prevalent lower extremity and back pain in adolescents corresponds with the findings by Rathleff et al. [14]. Lower extremity pain in children and adolescents has generally been related to growth and growth in combination with vigorous exercise [51]. Back pain has additionally often been related to habitual posture, lack of physical activity and variation in body movement [5, 52]. Head pain or headache however, has more commonly been associated to perceived stress and psychological conditions, also in children and adolescents [4, 17, 53]. The “head pain group” of this study also presents with the highest mean score of total stress (PSQ) (Table 3).

The mean stress level (PSQ-index mean score; 0.33 [SD 0.16]) of the study sample corresponds to previous studies in young people when applying the same stress instrument [39, 40], and the cut-off score for moderate to higher stress levels was set according to this. By using Bergdahl and Bergdahl [54] found a prevalence of high stress at 4.0 % in their Swedish the PSQ > 0.46 cut-off score, population based study (1275 subjects; 581 men and 694 women, aged 30+). In contrast, the prevalence of higher stress (PSQ ≥ 0.45) among the adolescents in this study was 22.0 %. In a general German population (2483 subjects; 1206 men and 1346 women, aged 18+), the prevalence of moderate to severe stress (PSQ ≥ 45) was 17.6 % [41]. Thus, the adolescents in this study had a greater prevalence of moderate to severe stress (PSQ ≥ 45) than what is previously found in other populations. The highest score among the adolescents was for demands (Table 1). This might indicate that coping with demands is a main stressor during adolescence, which is also discussed in Wiklund et al. [4]. Wiklund et al. [4] found that perceived stress in the form of pressure and demands were strongly correlated with health complaints and anxiety in Swedish older adolescents (16–18 years old). By more life experience, total stress perception seems to decrease [54].

The findings revealed significant associations between all factors of stress and pain measured in this study (Table 4). Higher levels of stress, including worries, tension, lack of joy and demands, correlated with more pain sites, longer pain duration and higher pain intensity (VAS). This might support the works of Murberg and Bru [17] and Eckhoff and Kvernmo [15], focusing on school-related stress. Looking at possible school-related stressors in a sample of 531 pupils aged 13–16 years, Murberg and Bru [18] found an association between these given stressors and psychosomatic symptoms. Eckhoff and Kvernmo [15] presented similar findings in a recent study based on The Norwegian Arctic Adolescent Health Study. They found school-related stress to be among the most important factors associated with musculoskeletal pain, and that musculoskeletal pain sites were associated with psychosocial problems.

The stress-pain association demonstrated in this study supports theories and basic research regarding the interconnection between these phenomena [11, 23, 24, 55]. The highest stress-pain correlation in the study appeared between stress (total PSQ and tension) and pain intensity (measured by VAS) (Table 4). With respect to previous studies using PSQ as a measure of stress, Kocalevent et al. [41] demonstrated significant correlations between stress and fatigue in a general German population. Mechanisms behind symptoms in fatigue and long-term pain conditions have been proved to be similar [56].

The results of the regression analyses imply that perceived stress (PSQ) was related to the reporting of musculoskeletal pain among the adolescents and probably explained some of the variation in pain intensity (VAS) and number of pain sites, controlled for gender and BMI (Tables 5 and 6). Naturally, the pain duration variable explained even more of the variation in pain intensity (VAS) and number of pain sites. When pain becomes persistent, sensitization mechanisms might be induced, resulting in a vicious circle where pain produces pain [9, 11, 56, 57]. However, the regression models do not explain all the variation in the dependent pain variables. The variation in pain might also be explained by other factors, e.g., genetic and social factors [21–24], physical/ sports activities, and injuries and diseases [14, 51, 52]. Studies based on the Young-HUNT study 2008 found that both chronic pain and psychological symptoms “runs in the family”, i.e., are most prevalent among adolescents with parents suffering from similar symptoms. [58, 59] Several authors have confirmed higher prevalence of musculoskeletal pain among girls [14, 16, 20, 60], suggesting gender to be an explanatory factor of pain. There was some higher prevalence of musculoskeletal pain among the girls also in this study sample (Table 1). However, girls also seem to be more prone to perceived stress [2, 4], suggesting stress to be a possible mediating factor of pain across genders. However, this was beyond the scope of this study.

The correlation between pain intensity (VAS) and tension can also be interpreted in the view of the modified transactional model of stress [26, 27]. The different factors of stress in this model correspond to the factors of stress in the PSQ, where tension represent the stress reaction. As the stress reaction has the potential to modulate or inhibit pain through the cortisol awakening response, the associated high pain intensity might indicate a deficiency in these mechanisms. The stress response might reflect a sustained and catabolic state of arousal [7], a chronic stress response possibly involving cortisol dysfunction [11, 28]. The stress response appears to no longer have a pain modulating/inhibiting function. Together with the association between pain intensity (VAS), pain duration, and pain sites (Table 3), this might imply that stress-induced pain sensitization [57] is present among the adolescents.

There were no significant mean differences between groups of adolescents with various types of musculoskeletal pain (Table 3 and 7). For instance, the neck/shoulder pain group presented with equivalent levels of stress as the extremity pain group. This contradicts the traditional view, considering neck and shoulder pain as more stress-related than extremity pain [3, 4, 13, 17]. This assumption resulted in an increased focus on cognitive-behavioral treatments for the presumed stress-related musculoskeletal pain conditions, showing a lack of success [61, 62]. The findings in this study underscores the importance of a comprehensive understanding of pain mechanisms in young people, as well as more appropriate and effective treatment and prevention approaches for this group. The group with head pain presented with the highest mean values of stress and pain variables in this study, though not significantly different (Table 3 and 7). Pairwise comparisons between pain groups including head or back pain, and the group of extremity pain exclusively, revealed small to medium effects sizes. The largest effect (0.5) appeared for (lack of) joy, comparing head and extremity pain. This might indicate that head pain affect happiness to some higher degree than extremity pain. In adolescents, head pain is often associated with other conditions such as anxiety and mood disorders [63] [64].

### Clinical implications

This study suggests that perceived stress (measured by the PSQ) can explain some of the probability of pain and variation in pain intensity and number of pain sites in adolescents (Tables 5 and 6). The findings shed some light on plausible mechanisms behind pain and illness in young people. Increased knowledge about this is crucial in order to succeed in treatment and health promotion among young people [38]. The demonstrated corresponding levels of stress (PSQ) between different types of musculoskeletal pain might also be clinical interesting. The lack of correlation between levels of stress and different types of musculoskeletal pain challenges common clinical understanding in that a painful knee could be considered just as affected by stress-mechanisms as neck and shoulder pain (Table 7). The association between pain intensity (VAS) and tension (stress-reaction) (Table 4), implies possible stress-induced sensitization among adolescents. If we increase stress and/or pain by our clinical methods and approaches, e.g., by intrusive techniques or highly demanding exercises, it might worsen and prolong pain- and stress-sensitized conditions and lead to more illness among the young people [11, 56].

### Strengths and limitations

The study investigated critical health parameters in adolescents, i.e., perceived stress and pain, which previously has been lacking [36, 38]. The findings shed new light on stress-pain associations in young people and provide a basis for further research. The applied stress instrument (PSQ) has several advantages, identifying direct effects of resources on the stress perception and indirect effects on health-related aspects [26, 27]. PSQ contains both positively and negatively formulated items in order to reduce acquiescent bias [44]. Similar to all questionnaire-based surveys, this study might be subject to potential self-reporting bias. Applying a relatively large sample size and powerful methods, these possible bias effects might be diminished. The items concerning pain-related injuries/ diseases were excluded for analyses, due to apparently very inconsistent comprehension of these concepts among the participants. In future studies this items can be specified, e.g., by defining alternative diagnosis/ conditions to choose between. However modern pain science focuses less on locale pain structures and local causes, especially in long-term pain conditions [11, 35, 55, 56]. The mechanisms behind the persistent and exhausting pain, on a “supersystem”-level, is considered more relevant and important [11, 22, 23]. Nevertheless, the cross-sectional design does not allow us to determine causal direction among the variables. The associations between stress and pain do not reveal whether the adolescents are stressed because of pain, or have pain because of stress. Further studies are needed to compliment the findings from this study, including more focus on protective health resources and gender differences among adolescents.

## Conclusion

Perceived stress and musculoskeletal pain are significantly associated in adolescents, according to the findings in this study. Different aspects of pain, i.e., number of pain sites, pain duration and pain intensity were significantly correlated with stress measured by different factors, i.e., worries, tension, joy and demands. Perceived stress (PSQ) was related to the reporting of musculoskeletal pain among the adolescents and could explain some of the variation in pain intensity (VAS) and number of pain sites. There were no significant differences in stress between various types of musculoskeletal pain, suggesting that stress-mechanisms are relevant in diverse musculoskeletal pain conditions in young people.

## Additional files

Additional file 1:
**STROBE checklist.** (80.0 KB) Additional file 2:
**Invitasjon til deltagelse i spørreundersøkelse for 10. trinn i Trondheim kommune.** (23.2 KB)

## Abbreviations

PSQ: Perceived stress questionnaire
Mod.PSQ: Modified Perceived stress questionnaire-index.
VAS: Visual analogue scale
SD: Standard deviation
CI: Confidence interval
IASP: International Association for the Study of Pain
BMI: Body mass index
GLM: General linear model
OR: Odds Ratio
W: Worries
T: Tension
J: Joy
D: Demands
