# Supplementary material for: Perceived stress and musculoskeletal pain are prevalent and significantly associated in adolescents: an epidemiological cross-sectional study
Source: BMC Public Health. 2015 Oct 23;15:1081. doi: 10.1186/s12889-015-2414-x (PMC4619092; doi:10.1186/s12889-015-2414-x)
Supplement: Additional file 2: — Invitasjon til deltagelse i spørreundersøkelse for 10. trinn i Trondheim kommune. (23.2 KB) [file 12889_2015_2414_MOESM2_ESM.docx]

**Invitasjon til deltagelse i spørreundersøkelse**

**for 10. trinn i Trondheim kommune**

Dette er en frivillig og anonym spørreundersøkelse. Den inngår i en studie om folkehelse blant norske ungdommer. Din deltagelse er av stor verdi og er med og danner grunnlag for videre folkehelsearbeid blant barn og unge. I den siste Folkehelsemeldingen: «God helse – felles ansvar» varsles en økt satsing på det forebyggende helsearbeidet i skolen og skolehelsetjenesten. For at forebyggende tiltak skal virke, er det avgjørende at relevante forhold blir kartlagt i forkant. Denne korte undersøkelsen er et ledd i dette.

Med vennlig hilsen,

Berit Østerås

Fysioterapeut, Msc, høgskolelektor

Høgskolen i Sør-Trøndelag

Avdeling for helse- og sosialfag

Program for fysioterapeututdanning

Del 1.

Kryss av i rutene og svar ellers som anvist.

**Dato: _________**

**JENTE** **GUTT**

1. Kjønn: □ □
2. Høyde: ______
3. Vekt: ______

**JA NEI**

1. Er du plaget med smerter? □ □

Hvis ja:

1. Hvor har du smerter?

Hode: □

Nakke: □

Skulder: □

Rygg: □

Arm: □

Bein: □ Eventuelt andre steder: _____________________

**JA** **NEI**

1. Har du/ har du hatt skade i det området hvor du nå har smerter? □ □

Eventuelt hvilken type: _______________________________

**JA** **NEI**

1. Har du kjent sykdom som er årsak til smertene? □ □

Eventuelt hva: _______________________________________

1. Hvor sterke er smertene dine? Kryss av på linja nedenfor for å markere din gjennomsnittlige smerte den siste uka. 0 = ingen smerte, 10 = verst tenkelige smerte.

0 10

1. Hvor lenge har du hatt disse plagene:

0-2 uker: □

2-4 uker: □

1-3 måneder: □

3 måneder eller mer: □

Del 2.

Sett ring rundt tallet som beskriver hvordan det er eller har vært for deg *den siste måneden*. Gjør dette raskt uten å sjekke svarene nøye og merk at det skal gjelde *den siste måneden*.

Nesten Av Ofte Vanligvis aldri og til

1. Du føler deg uthvilt 1 2 3 4

2. Du føler at du får for mange krav stilt til deg 1 2 3 4

3. Du er irritabel og gretten 1 2 3 4

4. Du har for mye å gjøre 1 2 3 4

5. Du føler deg ensom og isolert 1 2 3 4

6. Du opplever å være i konfliktsituasjoner 1 2 3 4

7. Du føler at du gjør ting som du virkelig liker 1 2 3 4

8. Du kjenner deg trøtt 1 2 3 4

9. Du frykter at du kanskje ikke klarer å nå

målene dine 1 2 3 4

10. Du føler deg rolig 1 2 3 4

11. Du har for mange avgjørelser å ta 1 2 3 4

12. Du føler deg frustrert 1 2 3 4

13. Du er full av energi 1 2 3 4

14. Du føler deg anspent 1 2 3 4

15. Problemene dine virker til å hope seg opp 1 2 3 4

16. Du føler at du har det travelt 1 2 3 4

17. Du føler deg trygg og beskyttet 1 2 3 4

18. Du har mange bekymringer 1 2 3 4

19. Du er under press fra andre mennesker 1 2 3 4

20. Du føler deg motløs 1 2 3 4

21. Du har det hyggelig 1 2 3 4

22. Du er redd for fremtiden 1 2 3 4

23. Du føler at du gjør ting fordi du må, ikke

fordi du vil 1 2 3 4

24. Du føler deg kritisert eller bedømt 1 2 3 4

25. Du er munter 1 2 3 4

26. Du føler deg mentalt utmattet 1 2 3 4

27. Du har problemer med å slappe av 1 2 3 4

28. Du føler deg tynget av ansvar 1 2 3 4

29. Du har nok tid til deg selv 1 2 3 4

30. Du føler deg presset av tidsfrister 1 2 3 4

**TUSEN TAKK FOR BESVARELSEN ☺**
